# The relationship between general practice characteristics, case-mix, and secondary care attendances/admissions before and after the COVID-19 pandemic: Protocol for an OpenSAFELY cohort study

**DOI:** 10.12688/wellcomeopenres.24356.1

**Published:** 2025-07-31

**Authors:** Mengxuan Zou, Shrinkhala Dawadi, Luisa M Pettigrew, Rosalind M Eggo, Emily Herrett, Venexia Walker, Michael Marks, Jonathan Sterne, Alex Walker, Arina Tamborska, Jaidip Gill, John Macleod, Johnny Filipe, Heather Mah, Sebastian Bacon, Matt Curtis, Amir Mehrkar, Ruth Costello, Rachel Denholm

**Affiliations:** 1 Bristol Medical School, Population Health Sciences, University of Bristol, Bristol, England, BS8 2BN, UK; 2 London School of Hygiene & Tropical Medicine, London, England, WC1E 7HT, UK; 3 Medical Research Council Integrative Epidemiology Unit, University of Bristol, Bristol, UK; 4 Department of Surgery, University of Pennsylvania Perelman School of Medicine, Philadelphia, USA; 5 Hospital for Tropical Diseases, University College London Hospital, London, UK; 6 NIHR Bristol Biomedical Research Centre, Bristol, England, UK; 7 Health Data Research UK South West, Bristol, UK; 8 Bennett Institute for Applied Data Science, University of Oxford, Oxford, UK; 9 The Phoenix Partnership, TPP House, Leeds, LS19 5PX, UK

**Keywords:** General practice characteristics; hospital admissions; A&E attendances; Ambulatory care; ACSC; OpenSAFELY.

## Abstract

**Background:**

Healthcare services in England experience increased pressure during winter months due to seasonal infectious diseases, increased multimorbidity, and fluctuating demand. Understanding how characteristics of general practices, and their registered patient case-mix contribute to secondary care use—particularly for Ambulatory Care Sensitive Conditions (ACSCs)—is essential for planning and resource allocation. Primary and secondary care activity also significantly changed during the COVID-19 pandemic, and not all activity-types have returned to pre-pandemic levels in the years since, making it critical to examine trends across both pre-and post-pandemic periods.

**Methods:**

OpenSAFELY-TPP was used to access linked electronic health record data, covering approximately 2,600 general practices (about 40% of all practices in England) and 26 million registered patients in England using TPP SystmOne software (2018-2025). Our analysis focused on weekly and aggregated rates of A&E attendances and hospital admissions during the flu and winter months (October to February), comparing patterns before and after the COVID-19 pandemic. Practice-level exposures included consultation rate per capita, practice size, region, and patient case-mix variables (e.g. age, sex, ethnicity, deprivation, multimorbidity). Outcomes included weekly rates of A&E attendances, total hospital admissions, and admissions for ACSCs.

**Analyses:**

We will summarise variation in practice characteristics, registered patient sociodemographics, case-mix, and service use across time periods. Associations between exposures and outcomes will be examined using generalised linear models, with additional subgroup analyses by age distribution. Sensitivity analyses will assess alternative flu season definitions and account for holiday and extreme weather.

**Discussion:**

This high-level descriptive study will provide valuable insights into variation in secondary care use across general practices and identify practice-level and case-mix factors that may contribute to winter pressures. The inclusion of both pre- and post-pandemic data will provide essential benchmarking data for future health system planning and further understanding of how the general practice context and patient case-mix affects hospital demand.

## Background and rationale

Primary and secondary care services in England experience increased pressures during the winter. Multiple factors can contribute to these pressures, including seasonal epidemics of respiratory infectious diseases such as COVID-19, influenza (flu), and respiratory syncytial virus (RSV) ^1^, increased cardiovascular disease incidence ^2, 3^ and the increasing healthcare needs of a growing and ageing population with multiple long-term conditions ^4^. However, despite the increasing demand, the capacity of primary care has not scaled correspondingly due to a decrease in qualified workforce numbers between 2015 and 2022 ^5^.

Pressures at the primary and secondary care levels are likely interrelated, with heightened primary care pressures and unmet demand having downstream consequences in secondary care, potentially significantly impacting resources, costs, and the quality of secondary care services. In England, unplanned hospital admissions account for 67% of hospital bed days and cost £12.5 billion annually (Department of Health, 2013) ^6^. Managing the demand for unplanned admissions has become a critical policy priority, with general practice playing a vital role in mitigating this burden.

Ambulatory Care Sensitive Conditions (ACSCs) are conditions for which timely and effective primary care can prevent hospital admissions, for example: asthma, diabetes, chronic obstructive pulmonary disease (COPD), hypertension, and angina ^7^. There is considerable variability in ACSC admission rates across general practices, even after accounting for patient demographics and chronic disease prevalence ^8, 9^. This suggests that practice-level factors, such as consultation provision rates, list size, region and rurality, and area-level deprivation may significantly influence secondary care utilisation ^10^.

Existing evidence highlights that practice-level characteristics (such as consultation provision rate) and patient case-mix (such as age), may be associated with variations in ACSCs admissions ^11, 12^. Describing the association between these practice characteristics and secondary care admissions is critical for developing strategies to manage demand and reduce pressures across the healthcare system.

## Aim

To investigate the relationship between general practice-level characteristics, including practice characteristics, patient sociodemographics and case-mix, and registered patients' use of secondary care services, and if this varies over time by ACSC and patient age.

## Research questions

1. Are there variations in weekly hospital attendance/admission across general practices during the flu and winter months (October - February) pre-pandemic (2018–2019) and post-pandemic (2022–2024)?
2. Are general practice characteristics and registered patient sociodemographics & case-mix associated with the weekly rate of hospital attendance/admission during the flu and winter months? Have these associations changed over time in the pre-pandemic (2018–2019) and post-pandemic periods (2022–2024), or by patients’ age?
3. Are the above characteristics and case-mix associated with the rate of hospital attendance/admissions for ACSCs (asthma, diabetes, COPD (including bronchitis), hypertension, and angina) vs non ACSCs during the flu and winter months, and have these associations changed over the pre-pandemic and post-pandemic periods, or by patients’ age?

## Methods

### Data sources

This study will use general practice electronic health records (EHRs) accessed via the OpenSAFELY-TPP platform. General practice data will be linked to the following data:

- Secondary Uses Services (SUS)
  - SUS Admitted Patient Care
  - SUS Emergency Care
- Office of National Statistics (ONS) death registry
- Second Generation Surveillance System (SGSS)
- Index of Multiple Deprivation (IMD) - patient-level

### Study population

Our study population consists of approximately 2,600 general practices in England (with about 26 million registered patients) between 2018 and 2024. This represents about 40% of general practices in England, and 42.6% of England’s population ^13^. All practices providing EHR data will be included in the analysis (i.e. regardless of whether they close or merge during the study period), as this is a descriptive study aiming to provide snapshots of real-world patterns in general practice characteristics and case-mix, and secondary care attendances/admissions. Patients will be included if they are alive and registered in an English general practice using the EHR software provider TPP at the start of each study period.

### Timeframes and cohorts

We will describe the data for the time periods before and after the COVID-19 pandemic, as the pandemic caused significant disruptions to general practice consultations ^14^ and secondary care attendances/admissions ^15, 16^:

- Pre-pandemic: January 2018 to March 2020
- Post-pandemic: April 2022 to March 2024

We will assess the associations between general practice characteristics and secondary care admissions/attendances over 5 months, encompassing the flu and winter seasons (October to February, inclusive). Full details of the overall study periods for each cohort, including the specific timeframes for exposure and outcome variables, are provided in Table 1.

**Table 1.** Summary of cohorts and study-period definitions.

	*Pre-pandemic, cohort 1*	*Post-pandemic, cohort 2*	*Post-pandemic, cohort 3*
Cohort start date	1 Oct 2018	1 Oct 2022	1 Oct 2023
Cohort end date	28 Feb 2019	28 Feb 2023	29 Feb 2024
***Exposure variables: practice-level characteristics & registered patient case-mix***			
Consultation provision rate: Total consultations per 1,000 registered patients at the practice	**Numerator:** Monthly count of consultations **Denominator:** Practice list size at the start of each month **Time period:** 12 months before each cohort start date; monthly rates calculated and then aggregated to a yearly rate.		
% of eligible patients who are vaccinated for: – Flu – COVID – Pneumococcal	**Numerator:** Count of registered patients who received the relevant vaccination (flu, COVID, or pneumococcal) in the 1-year period before the cohort start date. **Denominator:** Count of patients eligible* for the relevant vaccination in the 1-year period before the cohort start date. *Eligibility for free vaccination in general practice: • Flu: Aged ≥65 years, aged 2–3 years, or pregnant • COVID: eligibility criteria will depend on cohort, as guidelines have changed over time. • Pneumococcal: Aged ≥65 years **Time period:** One year before each cohort start date.		
Patient list size; Practice geographic region;	**Numerator:** Count of registered patients with each characteristic at the cohort start date. **Denominator:** Practice list size at the cohort start date. **Time period:** At cohort start date (i.e. as of October 1).		
% registered patients: - Female; - Age (0–5, 5–15, 65–75, 75–85, ≥85 years); - Ethnicity (White, Black, Asian, Other/Mixed) - Current smokers; - Obese (BMI >30); - Reside in each IMD quintile, according to their postcode; - That reside in an urban or rural area, according to their postcode			
Average Cambridge Multimorbidity score (CMS) per practice ^17^	**Numerator:** Sum of individual CMS scores for registered patients aged ≥20 years at the cohort start date. **Denominator:** Total number of registered patients aged ≥20 years in the practice at the cohort start date. **Time period:** At cohort start date (i.e. as of October 1).		
***Outcome: Weekly rate of A&E attendances and hospital admissions during flu & winter months or, for a more aggregated description, the overall winter-month rate.***			
Weekly rate of A&E attendances per 100,000 registered patients at the practice	**Numerator:** Weekly counts of A&E attendances or hospital admissions **Denominator:** Practice list size at the start of each week **Time period:** From each cohort start date to end date (i.e. the flu and winter months, October–February); calculated as a weekly rate over this period.		
Weekly rate of hospital admissions per 100,000 registered patients at the practice			
***Outcome: Weekly rate of A&E attendances and hospital admissions in flu/Winter months for ACSCs or, for a more aggregated description, the overall winter-month rate for ACSCs***			
Weekly rate of A&E attendances for ACSCs per 100,000 registered patients at the practice	Same definition and structure as the primary outcomes, but will be restricted to the primary five Ambulatory Care Sensitive Conditions (ACSCs): • Asthma • Chronic Obstructive Pulmonary Disease (COPD) • Diabetes • Hypertension • Angina **Time period:** From each cohort start date to end date (i.e. the flu and winter months, October–February); calculated as a weekly rate over this period.		

### Exposure variable definitions

All the exposure variables will be regarded as time in-variant and assessed either on or before the flu and winter months (as detailed in Table 1), depending on the required observation timeframes. Definitions for each exposure variable are provided in Table 2, encompassing both practice-level characteristics and registered patient sociodemographic and clinical case-mix. A summary is provided below:

**Table 2.** Exposures and data sources.

Exposures	Definition	Data sources (see *Extended data*) ^18^
***Practice characteristics***		
Practice geographic region	North East; North West; Yorkshire and The Humber; East Midlands; West Midlands; East of England; London; South East; South West.	Primary care
Practice list size	Total number of patients registered at the practice.	Primary care
Consultation provision rate	The monthly consultations at each practice, per 1000 registered patients, summed over 12 months prior to the flu & winter months.	Primary care
*Practice-level registered patient sociodemographics and case-mix*		
Sex	Proportion of registered patients by sex: male, female, unknown.	Primary care
Age	Proportion of patients in each age band: 0–5, 5–15, 65+, 75+, 85+ years, unknown.	Primary care
Ethnicity	Proportion of patients classified as White, Mixed, Asian or Asian British, Black or Black British, Chinese or Other, Unknown.	SNOMED
Deprivation	Proportion of patients living in each IMD deprivation quintile: 1 (most deprived), 2, 3, 4, 5 (least deprived), unknown; median IMD quintile of registered patients may also be considered	Index of multiple deprivation
Rurality	Proportion of patients living in each rurality classification: 1 (Urban major conurbation), 2 (Urban minor conurbation), 3 (Urban city and town), 4 (Urban city and town in a sparse setting), 5 (Rural town and fringe), 6 (Rural town and fringe in a sparse setting), 7 (Rural village and dispersed), 8 (Rural village and dispersed in a sparse setting), unknown.	Primary care
Current smoker proportion	Proportion of patients recorded as current smokers	CTV3
Obesity proportion	Proportion of patients with a diagnosis of obesity or BMI >30	SNOMED, ICD10
Cambridge Multimorbidity Score (CMS) - mean CMS per patient in each practice	**Take the average score** across all registered patients within each GP practice using 20 conditions: 1. Hypertension 2. Anxiety/depression 3. Painful condition (Osteoarthritis) 4. Hearing loss 5. Irritable bowel syndrome 6. Asthma 7. Diabetes mellitus 8. Coronary heart disease 9. Chronic kidney disease 10. Atrial fibrillation 11. Constipation 12. Stroke/transient ischemic attack (TIA) 13. COPD 14. Connective tissue disorder 15. Cancer 16. Alcohol problems 17. Heart failure 18. Dementia 19. Psychosis/bipolar disorder 20. Epilepsy $\begin{array}{l} Practice\;Level\;CMS\;=\\ \frac{\Sigma (Individual\;CMS\;for\;registered\;patients\;\geq \;20\;years\;old)}{Total\;registered\;patients\;\geq \;20\;years\;old\;in\;practice} \end{array}$	SNOMED
Vaccination - Flu	Proportion of eligible registered patients who received the vaccine in primary care for the year prior to flu & winter months	SNOMED flu vaccination codes
Vaccination - COVID-19	Pre-pandemic: Variable will not be defined Post-pandemic: Proportion of eligible registered patients who received **≥2 doses during pandemic** and **≥1 dose after 2022**, in line with UK vaccination guidelines introduced after 2022.	SNOMED COVID-19 vaccination codes

Note: All exposure variables are continuous, except for *practice region* (categorical) and *practice list size* (count variable).

**Practice-level characteristics** consist of the region of practice location (nine regions defined using the International Territorial Level 1 region of England), registered patient list size, and consultation provision rate. Patient list size is defined at the start of the time period specified in Table 1, and is used as the denominator for all practice-level measures to facilitate comparisons across practices. Consultation provision rate will be calculated for the full year preceding the flu and winter months, to reflect overall practice activity.

**Registered patient sociodemographic variables** are calculated as proportions of the total registered patient population within each practice at the start of the flu and winter months. These include the proportion of:

- Female patients (sex);
- Patients aged 0 to ≤5 years, 5 to ≤15 years, 65 to ≤75, 75 to ≤85, and ≥85 years (age);
- Patients classified as White, Mixed, Asian or Asian British, Black or Black British, Chinese or Other Ethnic Groups (ethnicity);
- Patients residing in different socioeconomically deprived quintiles, as defined by the Index of Multiple Deprivation (IMD; deprivation);
- Patients living in urban or rural areas, based on rurality classification.

Depending on the distribution of the data, deprivation may also be summarised using the median IMD quintile of registered patients within each practice. Similarly, rurality may be converted from a proportion-based measure to a binary categorical variable (urban/rural) if most patients within a practice share the same classification. The proportion of patients with missing data on sex, age, ethnicity, deprivation (IMD), or rurality will be reported to assess the completeness of sociodemographic information across practices.

**Registered patient case-mix variables** are also expressed as proportions of the total registered patients at each practice. These include:

- The proportion of current smokers
- The proportion of patients with a BMI >30 or a recorded diagnosis of obesity.
- The multimorbidity burden, which is quantified using the mean (or median, as appropriate) Cambridge Multimorbidity Score (CMS) per patient within each practice. The CMS is a validated, weighted index capturing disease burden based on the presence of multiple long-term conditions. It was derived using a large UK-based primary care cohort (n=300,000) and has been recently validated in two additional UK datasets ^17^.

### Outcome variable definitions

This study will examine weekly and aggregated rates of secondary care use during flu and winter months (October to February) in relation to general practice characteristics and patient case-mix.

***Research questions 1 & 2***

The primary outcomes for research questions 1 and 2 are the weekly rates of A&E attendances and hospital admissions during the flu and winter months (October to February). Aggregated winter-month rates may also be considered. Rates will be reported per 100,000 registered patients at the general practice and derived using:

- A&E attendances: Emergency Care Data Set (ECDS)
- Hospital admissions: Admitted Patient Care (APC) dataset.

$$\text{rate}\;=\;\frac{Number\;of\;attendances\;or\;admissions}{Registered\;patients\;at\;the\;practice}\;\;\times \;\;100,000$$

***Research question 3***

The secondary outcomes, aligned with research question 3, are weekly rates of A&E attendance and hospital admissions specifically for selected ACSCs: asthma, diabetes, COPD (including bronchitis), hypertension, and angina ( Table 3). Aggregated winter-month rates will also be considered.

**Table 3.** Outcomes and data sources.

*Outcomes*	*Secondary care (ICD-10, see Extended data)* ^18^
Asthma	ICD10: asthma
COPD, including bronchitis	ICD10: COPD
Diabetes	ICD10: diabetes
Hypertension	ICD10: hypertension
Angina	ICD10: angina

Note: For each ACSC, the outcome is defined as the weekly rate of A&E attendances or hospital admissions for the condition, per 100,000 person-weeks at the practice level. All outcome variables are continuous.

### Statistical analysis

***Research question 1***

Research question 1 will describe variation in hospital attendance and admission rates across practices. We will summarise general practice characteristics, including registered patient sociodemographics and case-mix. These characteristics will be described at the start of each flu and winter period. Median and interquartile ranges or means with standard deviations will be presented, as appropriate. Missing data will also be summarised for each exposure variable. Primary care consultation provision rates (defined in Table 1) will be presented overall and stratified by patient characteristics (e.g. age bands, sex, CMS quintiles) and practice-level factors (e.g. region).

Secondary care rates (defined in Table 1) will be described over the entire flu and winter months to illustrate patterns and variations over time. Rates for hospital admissions and A&E attendances will be presented separately; hospital admission rates will be further categorised by ACSC or non-ACSCs admissions.

We will additionally describe the distribution of general practice characteristics for practices with and without ACSC-related admissions. This output will illustrate how these characteristics differ by ACSC and non-ACSC admissions (see Table S1 in *Extended data*) ^18^.

***Research question 2***

To address research question 2, we will examine the associations between general practice-level characteristics, registered patient case-mix, and the rates of A&E attendance and hospital admissions (see Figure S1 in *Extended data*) ^18^. The choice of regression model will be informed by the distribution and shape of the outcome data. We will initially explore the suitability of linear models and, if necessary, evaluate alternative model forms such as Poisson or negative binomial models. We will also account for the correlation structure of our longitudinal outcome data by using general estimating equations (GEE). The final choice of model form will be selected based on the data structure and goodness-of-fit testing. As a sensitivity analysis to the main analysis, we will fit a mixed effects model to our data, to capture general practice-specific variability (e.g. through random effects).

***Research question 3***

To address research question 3, we will repeat the analyses described for research question 2 with ACSC-related hospital admissions and A&E attendances as outcomes, and separately for non-ACSCs. This will allow us to explore whether associations between practice characteristics and secondary care use differ for potentially avoidable admissions.

***Subgroup analysis***

We will conduct subgroup analyses restricted to practices with a high proportion of patients in specific age groups: 0 to ≤5 years; 5 to ≤15 years; 65 to ≤75 years; 75 to ≤85 years; and ≥85 years. High proportion will be defined empirically using the 95th percentile of the distribution across practices for each age group. These analyses will explore whether associations vary across practices with differing age compositions and will be conducted for the post-pandemic period only as more recent findings will have greater policy relevance for health system planning.

***Sensitivity analyses***

We will compare outcomes defined using our primary time window (October to February) with those based on the flu season as defined by the UK Health Security Agency (UKHSA), since the timing of flu activity can vary across years. This will allow us to evaluate whether our fixed study period adequately captures true seasonal flu activity, and whether any potential mismatch meaningfully affects observed rates of secondary care use.

We will also flag, and account for weeks affected by unstable external factors, such as extreme weather events or public holidays, which may influence secondary care use. Weeks with severe weather will be identified using the UK Met Office’s National Severe Weather Warning Service ( https://www.google.com/url?q=https://www.metoffice.gov.uk/research/library-and-archive/publications/national-severe-weather-warning-service).

### Patient and Public Involvement and advisory input

We formed a Patient and Public Involvement and Engagement (PPIE) advisory group to provide feedback throughout the duration of this project. The group comprised seven people from across England with lived experience of accessing primary and secondary care services, including some members with multiple long-term conditions. We first met online in February 2025 to discuss this research. The advisory group members provided helpful input based on their experiences. For example, they suggested accounting for the impact of severe weather events and holiday weekends in our study, which we have now incorporated into our sensitivity analyses. We paid the members for their time, following NIHR guidelines.

### Study status and dissemination

We are currently finalising data management and wrangling for this study. The study population has been defined, and all necessary variables have been created. Data checks are ongoing. We plan to begin the analysis described here during the week commencing 10 June 2025.

Data management has been carried out using ehrQL (Electronic Health Records Query Language; more information available at https://docs.opensafely.org/ehrql/), with analyses conducted using R version 4.5.0 and Stata version 19.0. All code is openly shared under an MIT open license to support transparency and reuse. Once the analyses described in this document are complete, all data management and analysis code will be archived at: https://github.com/opensafely/WinterPressuresDescriptive/. Clinical and medicines codelists used in this study are openly available at https://codelists.opensafely.org/.

## Discussion

This protocol outlines a large-scale, population-based cohort study designed to investigate the relationship between general practice characteristics, registered patient case-mix, and secondary care attendances and admissions during winter months, both before and after the COVID-19 pandemic. Leveraging the OpenSAFELY-TPP platform, we will examine data from approximately 2,600 general practices across England, representing over 40% of all practices and nearly 43% of the national population. This provides a unique opportunity to explore real-world variation in healthcare use and to generate evidence that may inform service planning and policy. These insights may also help to identify modifiable practice-level drivers of high hospital use, particularly for ACSCs, where timely and effective primary care has the potential to reduce avoidable admissions.

As this is not a patient-level cohort analysis, we have treated all exposure variables as time-invariant, measured on or before the start of each winter period. This approach reflects the assumption that key practice-level characteristics — such as patient age structure, multimorbidity burden, or smoking prevalence — are unlikely to change substantially in the short term. This allows for consistent comparisons across flu seasons while capturing meaningful differences in practice context and case-mix.

Although consultation provision rate is a practice-level measure, it is derived from patient-level data, and the composition of registered patients can fluctuate throughout the year as individuals join or leave practices. To reflect underlying practice activity rather than short-term fluctuations, while ensuring a stable estimate of routine activity, we will calculate monthly consultation provision rates (defined as the number of consultations per 1,000 registered patients each month) and aggregate them over the 12 months prior to each winter period. This approach balances temporal accuracy and stability, providing a more representative estimate of usual practice workload, and avoiding bias from short-term variation or seasonal spikes in demand.

To maximise sample size and preserve representativeness, we have not applied a 90-day prior registration requirement for patients. While such thresholds are often used in patient-level studies to ensure completeness of individual records following registration, our focus is on practice-level characteristics and demand. Excluding patients based on short registration durations could omit relevant workload pressures — particularly for times or areas of high population movement — and is therefore not necessary for this descriptive practice-level analysis.

A key strength of this study is the inclusion of both pre-pandemic and post-pandemic time periods. We have chosen to define the post-pandemic period as April 2022. Although the World Health Organization officially declared the end of the global COVID-19 public health emergency in May 2023, our earlier start date better reflects the situation in the UK. From February 2022, health services in the UK adopted a “living with COVID-19” policy, and service delivery in both general practice and hospitals began operating under more routine conditions ^19^. The COVID-19 pandemic may lead to substantial and lasting changes in both primary and secondary care ^20^. Defining one cohort for each post-pandemic year allows us to better describe and compare how the relationship between general practice context and secondary care use may have changed across the post-pandemic era. These findings will support a better understanding of how structural and case-mix factors contribute to winter pressures and inform future health service planning.

We have incorporated feedback on study design from our PPIE advisory group, for example, identifying relevant exposures and sensitivity analyses (such as accounting for extreme weather or holiday weeks). This feedback has helped ensure that our research questions and analytic approach are grounded in real-world experiences, and that our findings will be applicable to health policy and clinical practice. We will continue consulting with our advisory groups throughout the study.

In summary, we have outlined a protocol to explore the characterisation of variation in winter-time secondary care use across English general practices and identify potential practice-level drivers of higher or lower service demand. The results may support future interventions aimed at improving integration between primary and secondary care, reducing avoidable hospital use, and enhancing preparedness for seasonal healthcare pressures.

## Ethics and information governance

Ethics approval received from HRA and Health and Care Research Wales (HCRW) on 04 December 2024, REC reference: 24/HRA/5020.

## Data Availability

No underlying data are associated with this article. Zenodo: Extended_data_GP_characteristics_and_hospital_use_protocol_16072025.docx ^18^. https://doi.org/10.5281/zenodo.15983958 The extended data include: Codelist details for Table 1 and Table 2 Table S1. Descriptive statistics of practice level characteristics by ACSC and non-ACSC admission Figure S1. Forest plot of the associations between general practice characteristics and case-mix and A&E attendance and hospital admission (example) Data are available under the terms of the Creative Commons Attribution 4.0 International license (CC-BY 4.0).

## Author information

Authors Ruth Costello and Rachel Denholm jointly supervised this work, while Mengxuan Zou and Shrinkhala Dawadi contributed equally.
