# Supplementary material for: The relationship between general practice characteristics, case-mix, and secondary care attendances/admissions before and after the COVID-19 pandemic: Protocol for an OpenSAFELY cohort study
Source: Wellcome Open Res. 2025 Jul 31;10:396. [Version 1] doi: 10.12688/wellcomeopenres.24356.1 (PMC12831950; doi:10.12688/wellcomeopenres.24356.1)
Supplement: Peer review history

# Reviewer report: Reviewer response for version 1

**DOI:** 10.21956/wellcomeopenres.26857.r144071

**Published:** 2026-01-24

**Authors:** Ulrik Bak Kirk

**Affiliations:** 1 Aarhus University, Aarhus, Denmark

Thank you for the opportunity to review this protocol. This is a timely, well-constructed, and policy-relevant study that addresses a question of clear importance to health-system planning in England: how variation in general practice characteristics and registered patient case-mix relates to secondary care utilisation during winter pressures, across pre- and post-pandemic periods.

 The protocol is clearly written, methodologically robust, and makes excellent use of the OpenSAFELY-TPP platform. The rationale, data sources, exposure definitions, and analytic framework are described in detail to allow replication. The inclusion of both overall and ACSC-specific outcomes, and of pre- and post-pandemic cohorts, is a major strength. I also commend the authors for their clear commitment to transparency (open code, code lists, and analytic plans) and for the meaningful involvement of a PPIE advisory group.

 I have a small number of comments and suggestions that may help further clarify the study’s intent and enhance interpretability:

 1. While the abstract clearly conveys the context and methods, it would benefit from a more explicit statement of the overall study aim in a single sentence

 2. The rationale for including only one pre-pandemic winter (2018–19), compared with two post-pandemic winters, would benefit from clearer justification.

 3. The protocol would benefit from a slightly more explicit description of the analytic unit (practice-week observations) and how repeated weekly measures within practices and within winters are handled in the regression models.

 4. The intention to explore differences by age is appropriate, but there is some ambiguity in the phrasing of research questions 2 and 3.

 5. Although the descriptive and ecological nature of the analysis is implicit throughout, it would be helpful to state this more explicitly in the discussion.

 6. Given substantial variation in age structure between practices, the suggestion to consider presenting age-standardised descriptive rates alongside crude rates is sensible and would likely enhance interpretability of between-practice variation, even if regression models already adjust for age composition.

 7. Many exposure variables (age structure, multimorbidity, deprivation, smoking, obesity) are plausibly correlated. A brief statement on how multicollinearity will be assessed (e.g. variance inflation factors, correlation matrices) and how this will inform model specification would further strengthen the analysis plan.

 8. Clarifying how practice closures or mergers occurring during a winter period will be handled, particularly with respect to denominators and attribution of weekly outcomes, would improve the reproducibility and interpretability of rate calculations.

 9. The background refers primarily to unplanned admissions, while the methods refer more generally to hospital admissions. Clarifying whether admissions are restricted to emergency/unplanned admissions would help align background, rationale, and outcome definitions.

 10. The justification for using consultation provision rates aggregated over the preceding year is clear and reasonable. However, the discussion could briefly acknowledge that seasonal surges in consultation demand might themselves be of interest and could plausibly relate to acuity and downstream hospital use.

Is the study design appropriate for the research question?

Yes

Is the rationale for, and objectives of, the study clearly described?

Partly

Are sufficient details of the methods provided to allow replication by others?

Yes

Are the datasets clearly presented in a useable and accessible format?

Not applicable

Reviewer Expertise:

General practice, digital health, implementation

I confirm that I have read this submission and believe that I have an appropriate level of expertise to confirm that it is of an acceptable scientific standard, however I have significant reservations, as outlined above.

# Reviewer report: Reviewer response for version 1

**DOI:** 10.21956/wellcomeopenres.26857.r144078

**Published:** 2026-01-09

**Authors:** Catherine Atkin

**Affiliations:** 1 University of Birmingham, Birmingham, UK

This article describes a protocol for an analysis of primary and secondary care data, accessed using the Open_SAFELY platform, aiming to identify associations between practice level factors (at level of GP practice) and rates of hospital attendance and admission.

 The protocol provides a good background to the question, and overall provides a clear explanation of the rationale and intended analysis approach.

 There are a few points that could be improved or clarified:

- The objectives are explained, but there are research questions 2 &3 would benefit from some clarification. They both discuss comparison over time, which is clearly explained, however they say “Have these associations changed over time” “by patients’ age”. The explanation of this subgroup analysis in the statistical analysis section is clearer, and clarifies that this is not changes by patients age, but comparison between practices with high proportions of patients at extremes of age (i.e. practice level, not patient level; stratified, by age group). This should be explained more clearly in the research questions (or removed from the questions and discussed elsewhere).
- The abstract would benefit from clearly stating the overall aim of the study – it is alluded to, but not clearly stated.
- The dates for the study are explained as being divided into two time periods (pre and post pandemic), with pre-pandemic definition given as Jan 2018 to March 2020, however then there is only one pre-pandemic time period (Oct 2018-Feb 2019). Was there a reason why Oct 2019-Feb 2020 was not included, given that it falls within the defined ‘pre-pandemic’ period?
- The background discusses the rise in unplanned hospital admissions causing increased pressure. The remainder of the manuscript discusses ‘hospital admissions’. Is this restricted to unplanned/emergency admissions, or is this all admissions? If elective admissions are included, will this potentially cause underestimation of the increase in unplanned admissions (if elective care may decrease in times of increased pressure e.g. peaks of winter pressure)?
- It may be useful to clarify that when you are looking at admission reason, how this is defined, i.e. the primary diagnosis.
- The methods state *“We will additionally describe the distribution of general practice characteristics for practices with and without ACSC-related admissions* *”. *This doesn’t match the explanation in the following sentence, or the linked supplementary table, which discuss comparing practice level characteristics between non-ACSC and ACSC admissions. Is it comparison between practices, or comparison between admissions? If the comparison is between practices, then how will practices ‘without’ ACSC-related admission be defined? Is it likely that there will be practices with no ACSC related attendances or admissions (given that some admissions for asthma & COPD may not be completely avoidable).
- The background contains reference to a Department of Health figure (given in brackets), but this is not in the reference list.
- The methods has a PPI section, which is positive. As this is a protocol for the study, it would be useful to include some information about the planned PPI activity or engagement that will take place in the study, in addition to what PPI has already contributed.
- The discussion provides a fairly clear rationale for why consultation rates have been taken as an average over the year, but does say that this approach has partly been taken to avoid ‘seasonal spikes in demand’. Have you considered how the seasonal spikes in demand might be beneficial to consider, for example how increases in demand might be associated with changes in acuity of illness, resource availability to individual patients, and with increased secondary care use, e.g. emergency department attendances.

Is the study design appropriate for the research question?

Yes

Is the rationale for, and objectives of, the study clearly described?

Partly

Are sufficient details of the methods provided to allow replication by others?

Yes

Are the datasets clearly presented in a useable and accessible format?

Not applicable

Reviewer Expertise:

Acute medicine, particularly admission pathways for unplanned medical admissions. This includes use of routinely collected electronic health record data to assess acute care pathways within secondary care.

I confirm that I have read this submission and believe that I have an appropriate level of expertise to confirm that it is of an acceptable scientific standard, however I have significant reservations, as outlined above.

# Reviewer report: Reviewer response for version 1

**DOI:** 10.21956/wellcomeopenres.26857.r142662

**Published:** 2026-01-08

**Authors:** Moien Khan

**Affiliations:** 1 United Arab Emirates University, Al-Ain, United Arab Emirates

Thank you for the opportunity to review this protocol. It addresses an important and timely question, and the overall rationale, objectives, and use of the OpenSAFELY platform are clearly and convincingly presented. The study design is appropriate for the research questions, and the protocol is well aligned with current priorities around winter pressures and health-system planning.

 A few minor points may help to further enhance clarity and strengthen the protocol:

  

1. The rationale for examining both overall and ACSC‑specific attendances/admissions is clear and well justified, with good alignment between aims, research questions and planned analyses.
2. It would help readers if the choice of winter seasons were explained more fully, especially why only one pre‑pandemic winter (2018–19) is used compared with two post‑pandemic winters.
3. The unit of analysis and time structure for the regression models (practice‑week observations, correlation structure for GEE, handling of seasons) could be described a little more explicitly.
4. Given large differences in age structure between practices, the authors may wish to present age‑standardised descriptive rates alongside crude rates to aid interpretation of between‑practice variation.
5. Many exposure variables are likely to be correlated like age mix, multimorbidity, deprivation; a brief note on how multicollinearity will be checked and handled would strengthen the analysis plan.
6. The decision to treat practice‑level exposures as time‑invariant is reasonable, but it would be useful to emphasise that the study is primarily describing differences between practices rather than changes within practices.
7. Clarification on how practice closures and mergers during a winter period will be handled (for both numerators and denominators) would make the denominators and rate calculations easier to follow.
8. The ACSC code lists and outcome definitions are clearly referenced in the extended data; a short statement that these follow established definitions would reassure readers about comparability.
9. The representativeness paper for OpenSAFELY‑TPP is cited; summarising in one or two sentences how TPP practices compare with all English practices would help readers judge generalisability.
10. The description of PPIE is excellent; it might be worth highlighting in the abstract or discussion that patient/public input directly shaped sensitivity analyses like holiday periods and extreme weather.
11. The discussion could briefly restate that this is a descriptive, ecological analysis and that associations between practice characteristics and hospital use should not be interpreted causally but as hypothesis‑generating.

Is the study design appropriate for the research question?

Yes

Is the rationale for, and objectives of, the study clearly described?

Yes

Are sufficient details of the methods provided to allow replication by others?

Yes

Are the datasets clearly presented in a useable and accessible format?

Not applicable

Reviewer Expertise:

Health and Wellness, Primary Care , Global Health , Lifestyle medicine

I confirm that I have read this submission and believe that I have an appropriate level of expertise to confirm that it is of an acceptable scientific standard.
